# Effects of mid-to-late prepartum feed supplementation in Hanwoo beef cows on their performance, blood metabolites, and the carcass characteristics and metabolites of their neonatal calves

**DOI:** 10.3389/fvets.2023.1287119

**Published:** 2023-11-16

**Authors:** Myung Sun Park, Borhan Shokrollahi, Ui Hyung Kim, Jeong Il Won, Soo-Hyun Cho, Shil Jin, Sung Sik Kang, Sung Jin Moon, Kyung-Hwan Um, Ki Suk Jang, Hyoun Ju Kim, Nam Young Kim, Sung Woo Kim, Sun Sik Jang, Hyun-Jeong Lee

**Affiliations:** ^1^Hanwoo Research Institute, National Institute of Animal Science, Pyeongchang, Republic of Korea; ^2^Department of Animal Science, Sanandaj Branch, Islamic Azad University, Sanandaj, Kurdistan, Iran; ^3^Animal Products Utilization Division, National Institute of Animal Science, RDA, Wanju, Republic of Korea

**Keywords:** prepartum feed supplementation, Hanwoo beef cows, blood metabolites, carcass characteristics, neonatal calves

## Abstract

**Introduction:**

This study aimed to evaluate the implications of supplementary nutrition during the mid-to-late pregnancy on various parameters in Hanwoo cows and their subsequent neonatal calves.

**Materials and methods:**

Eight Hanwoo cows in their first parity were divided into two groups. The control group (C, 100%) received 3kg of concentrate and 5kg of rice straw throughout the pregnancy period, while the treatment group (T, 150%) increased their diet during mid-to-late pregnancy. Both performance assessments and blood metabolite analyses were performed for the pregnant cows. Neonatal calves were subjected to morphometric evaluations, blood sampling, and detailed morphometric analyses of carcasses and gastrointestinal components.

**Results:**

Performance indices of the cows showed that both Pregnancy Period (PregP) and Body Condition Score (BCS) were significantly improved with supplemental feeding (*p* <0.05). Improvements in Body Weight (BW) and Feed Conversion Ratio (FCR) were not statistically significant. Blood metabolite analysis for the cows revealed decreased levels of triglycerides (TGLate), non-esterified fatty acids (NEFALate), and progesterone (P4Late), with a notable increase in glucose (GluLate) levels (*p* <0.01). In the neonatal calves, anatomical metrics of the gastrointestinal tissues showed increased Omasum Width (OmasWdth) values in the supplemented group (*p* =0.053). There was significant increase of papillae and villus lengths in the rumen and small intestine (*p* <0.01 and *p* <0.05, respectively). Morphometric evaluations displayed longer body lengths (BLnth) and larger chest width (ChestWdth) in the treated calves (*p* <0.05 and *p* <0.01, respectively). Carcass characteristics showed no substantial variations between the groups, while blood analysis in the calves revealed decreased GPT levels in the nutritionally supplemented group (*p*<0.05).

**Discussion:**

The findings indicate that supplementing the diets of Hanwoo cows during mid-to-late pregnancy leads to significant changes in select maternal blood metabolites and influences specific anatomical and morphometric features in neonatal calves, all without significant shifts in carcass attributes.

## 1. Introduction

In the field of livestock development, maternal nutrition is of paramount importance. The patterns of nutrient consumption by the mother directly impact the physiological outcomes of the offspring. Nutritional status, especially during the mid-to-late pregnancy stages, plays a crucial role in regulating nutrient distribution in cows. This distribution critically influences the development and morphogenesis of the primary fetal organ systems (1, 2) and play a vital role in determining the quality of livestock yield. It’s well-recognized that progeny with a birth weight above the mean exhibit a higher survival rate in most livestock species (1). As the industry progresses, understanding this intricate relationship becomes increasingly crucial for producers striving to optimize herd health and beef quality as well as quantity (3–5). This not only affects muscle growth but is also pivotal for the formation of intramuscular adipocytes, which are essential for producing high-quality beef (6). The complex relationship between total fiber count and growth has been well-established (7, 8). While most postnatal muscle growth occurs through the enlargement of these fibers (9–11), the actual specification and quantity of muscle fibers are set during intrauterine development (12, 13). This underscores the importance of maternal nutrition during pregnancy, a period where nutritional intake can promote muscle fiber synthesis and growth, ultimately determining the quality of offspring meat (10, 14). On the other hand, adipose tissue in cattle not only acts as an energy reservoir but also has essential endocrine roles (15). It plays a significant role in determining the meat’s flavor, texture (16), and economic worth. In the case of Hanwoo beef, intramuscular fat is of utmost importance, significantly influencing more than 90% of the meat’s grading (17) and consumer choices. Both adipocytes and myocytes differentiate from mesenchymal stem cells, leading to potential competition during their formation (18). The onset of adiposeness (increasing fat cells) in cattle aligns with active secondary myogenesis during mid-pregnancy, and maternal diet shapes the muscle-to-fat proportion (19).

Recent studies postulate that maternal nutrition, particularly during the critical late pregnancy period, has the potential to bring about substantial changes in the carcass composition of offspring, resulting in variations in the yield and quality of red meat (2, 11, 20). This holds substantial implications in the field of beef production, where the objective is to maximize the performance of both the cow and the calf. To achieve these goals, rigorous nutritional strategies are necessary (21). Additionally, the role of maternal nutrition thus expands beyond immediate effects. According to the metabolic imprinting hypothesis, fetal alterations owing to maternal nutrition or endocrine changes can induce enduring modifications in fetal morphology and metabolic functions (22). These alterations can reflect into the postnatal life, impacting the offspring’s metabolism, physiology, and production capability (23).

For the beef industry, in particular in the context of Hanwoo beef—a breed celebrated for its distinctive marbling and high-quality meat—it’s imperative to evaluate the role of mother nutrition during pregnancy. The performance and metabolite indices of cows can be significantly influenced by their dietary regimes. Moreover, these nutritional factors have a cascading effect. They can influence not only the immediate offspring’s growth and muscle development but also their carcass characteristics and metabolites.

According to this foundational knowledge, this study aims to investigate the feed supplementation effect on Hanwoo beef cows during the mid-to-late pregnancy period. Initially, we assess maternal performance parameters and blood metabolite profiles, then analyze the influence on neonatal calf carcass characteristics and blood metabolite concentrations.

## 2. Materials and methods

### 2.1. Animals and experimental design and diets

This research was conducted over a duration of 10 months, from June 2021 to April 2022, at the Hanwoo Research Institute affiliated with the National Institute of Animal Science (NIAS). The study encompassed 8 Hanwoo cows, all in their first parity. Prior to artificial insemination, these cows were uniformly housed and were administered a standard diet of 3 kg of concentrate feed and 5 kg of rice straw. Following artificial insemination, the cows were divided into two distinct groups, each containing four individuals, based on their respective weights and ages. Their diet continued to include rice straw and the concentrate feed. The control group (C, 100%) consistently received 3 kg of concentrate and 5 kg of rice straw throughout the pregnancy period, starting from artificial insemination until parturition. In contrast, the treatment group (T, 150%) followed a dietary pattern identical to the control group during the early pregnancy phase (from artificial insemination to 3 months post-insemination). However, during the mid (3 to 6 months of pregnancy) and late pregnancy periods (6–9 months), their dietary provision was increased to 4.5 kg of concentrate and 6.5 kg of rice straw. A detailed analysis of the feed components was performed in compliance with the (24) analytical protocols. The chemical composition of the diets is detailed in Table 1.

**Table 1:** Chemical constituents of the experimental diets for Hanwoo cows.

Item^1^	Concentrate	Rice straw
Dry matter	88.43	97.87
% of dry matter		
Crude Protein	16.43	6.22
TDN	75.40	60.64
NDF	35.64	71.47
ADF	13.83	38.56
Ash	7.55	11.31
Ether extract	3.42	0.64
Crude fiber	8.14	29.99
Lignin	2.88	4.50

1 Total digestible nutrient (TDN); Neutral detergent fiber (NDF); Acid detergent fiber (ADF).

### 2.2. Cows management

The management of the experimental cows followed the guidelines set by the Hanwoo Research Institute, with feedings scheduled twice a day at 08: 00 and 16: 00. Throughout the day and night, the cows had *ad libitum* access to water and mineral blocks.

### 2.3. Performance assessments and blood sample collections from cows

Body weight (BW) and Body condition score (BCS) were measured at the early (month 2), mid (month 5) and late (month 8) stages of pregnancy. Feed conversion ratio (FCR) was assessed for early to mid (FCREarMid) and mid to late (FCRMidLate) stages of pregnancy. Blood samples were taken from experimental cows during the early, mid, and late periods of pregnancy. These were drawn from the jugular vein 3 h post morning feed. For each cow, 10 mL of blood was collected into a vacutainer and then transported to the laboratory. Within 6 h, serum was separated using a centrifuge at room temperature (20 min at 2,000 × g; Labogene, 1,580, Korea). Following this, blood metabolites were assessed using an automatic biochemistry analyzer. The studied blood metabolites included albumin (Alb, g/d), glucose (Glu, mg/dl), cholesterol (Cho, mg/dl), triglycerides (TG, mg/dl), blood urea nitrogen (BUN, mg/dl), total protein (TP, mg/dl), and non-esterified fatty acids (NEFA, μEq/l). Blood concentrations of estradiol-17β (E2) and progesterone (P4) were analyzed using an ELISA Kit from Abnova (Walnut, CA, United States). Each sample was assayed twice with an ELISA Multimode Plate Reader from Perkin Elmer Life Sciences (Waltham, MA, USA) set at a 450 nm wavelength

### 2.4. Neonatal calf slaughtering: blood sampling and morphological measurements of carcass and gastrointestinal components

Following parturition, neonatal calves were immediately separated from their maternal counterparts without receiving the colostrum. They were then subjected to morphometric evaluations, wherein parameters such as birth weight [BWght (kg)], height (BHght (cm)), and length [Blnth (cm)] were documented. Subsequent to these initial measurements, the calves were slaughtered. After slaughtering, blood samples were collected and expeditiously transported under controlled conditions to the laboratory. Within a period of 6 h post-collection, serum was systematically extracted from these samples, ensuring optimal preservation of biochemical constituents.

Detailed morphometric analyses of the carcasses were carried out. Specific anatomical regions including the rump (parameters: RumpHght, RumpLnth, RumpWdth), chest (parameters: ChestDpth, ChestWdth, ChestGrth), and thurl (parameter: ThurlWdth) were measured, encompassing dimensions such as height (cm), width (cm), depth (cm), girth (cm), and length (cm).

Further anatomical examinations delved into the gastrointestinal components. Organs, namely the liver, rumen, reticulum, omasum, abomasum, and small intestine, were meticulously assessed for their weight (kg), length (cm), and width (cm), denoted, respectively, as LiverWgth, LiverLnth, LiverWdth, and similar notations for each organ.

To evaluate the yield and quality of the carcass, the cold carcass weight was assessed. Subsequently, identification and separation of the main meat cuts (tenderloin (Tendloin), sirloin, striploin, chuck, plate, round (Round), bottom round (BotRound), shank, brisket, and rib) were carried out. Each cut was then quantitatively analyzed to determine its relative weight percentage compared to the total cold carcass weight. Finalizing the carcass evaluation, the relative weights of bone, meat, and adipose tissue were calculated.

### 2.5. Processing and histomorphometric analysis of muscle tissues

Samples were obtained from the sirloin, stomach (rumen, reticulum, omasum, and abomasum), and small intestine regions. Immediately after extraction, the samples were fixed in a 10% formaldehyde solution to preserve tissue morphology. After fixation, the tissues underwent sequential dehydration. The samples were subsequently embedded in paraffin wax using an automatic tissue processor (Leica TP1020, Semienclosed benchtop tissue processor, Leica Wetzlar, Germany). Tissue sections with a thickness of 4 μm were prepared and mounted on glass slides.

For immunohistochemistry, the sections underwent a deparaffinization process and were rehydrated. After that, they were rinsed with phosphate-buffered saline (PBS) and blocked using a solution of 2% bovine serum albumin (BSA) containing 0.3% Triton X-100. This blocking solution was allowed to incubate at room temperature for 1 h to reduce non-specific binding. Primary antibodies against hyaluronic acid, collagen I, and collagen III (all at a dilution of 1:400; sourced from Abcam, Cambridge, U.K.) were applied to the sections and incubated at 4°C for 24 h. After primary antibody incubation, the sections were thoroughly washed to remove any unbound antibodies. Goat anti-rabbit secondary antibodies (1:1,000) (ENZO Biochem, Farmingdale, NY, USA) were used for double staining purposes with 3,3-diaminobenzidine (DAB). The sections were also weakly stained with hematoxylin for nuclear staining. The incubation for this step was done at room temperature for 1 h. To ensure the specificity of our staining protocol, a negative control was included. In this control, the primary antibody was replaced with PBS containing 1% BSA.

Histomorphometric analysis was conducted to assess parameters such as the length and count of papillae and villi in the rumen, reticulum, omasum, and small intestine. Additionally, we evaluated the thickness of the abomasum (labeled as AbomMusThick) as well as the diameter and count of muscle fibers in loin and rump muscle samples from each stained section (representative images are shown in Supplementary Figures).

### 2.6. Blood metabolites analysis of claves

In the blood samples of slaughtered neonatal calves, the primary components measured were albumin (ALB, g/dL), glucose (GLU, mg/dL), cholesterol (CHO, mg/dL), triglycerides (TG, mg/dL), blood urea nitrogen (BUN, mg/dL), total protein (TP, mg/dL), Glutamate Oxaloacetate Transaminase (GOT), Glutamate Pyruvate Transaminase (GPT), phosphorous (P), and non-esterified fatty acids (NEFA, μEq/l).

### 2.7. Statistical analysis

All data associated with various variables were analyzed using the *t*-test procedure in SAS (version 9.4; SAS Institute Inc., Cary, NC, United States). The aim was to assess the effects of supplemental nutrition during the mid-to-late stages of pregnancy on different parameters in Hanwoo cows and their slaughtered male calves. For the cows, measurements of specific body parameters (such as BW and BCS during the middle, late, and post-pregnancy periods) and blood metabolites (like albumin, glucose, cholesterol, triglycerides, blood urea nitrogen, total protein, non-esterified fatty acids, estradiol, and progesterone during the middle and late pregnancy periods) were adjusted based on their early measurements, which were used as covariates. The data for the slaughtered calves were similarly analyzed using the t-test procedure in SAS. Correlations between the various parameters of the cows and their slaughtered calves were then analyzed and visualized using R (version 4.3.1).

## 3. Results

### 3.1. Effects of supplemental nutrition during mid-to-late pregnancy on performance indices and blood metabolites in Hanwoo cows

#### 3.1.1. Performance indices

**Figure 1:**
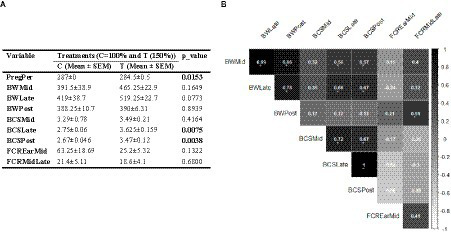
**(A)** Influence of nutritional supplementation during mid-to-late pregnancy on cow performance indices. **(B)** Correlation between pairs of these performance metrics. Correlation coefficient values higher than 0.5 are marked with an “*” sign.

The results revealed that both PregP and BCS significantly increased in Hanwoo pregnant cows that received supplemental feeding compared to those in the control group (Figure 1A; *p* < 0.05). In contrast, while BW and FCR measures showed improvement in the treated cows, these changes were not statistically significant. Correlation coefficient analysis indicated that BW during pregnancy had moderate to high correlations with most other traits, except for FCREarMid. However, this trend was not observed for BWPost. Additionally, there was negative correlations between BCSs and FCRs (Figure 1B).

#### 3.1.2. Blood metabolites

**Figure 2:**
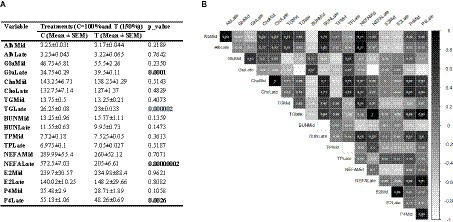
**(A)** Effect of nutritional supplementation during mid-to-late pregnancy on cow metabolite parameters. **(B)** Correlation between different pairs of these metabolic parameters. Correlation coefficient values higher than 0.5 are marked with an “*” sign.

The results from the blood metabolite analysis indicate that TGLate, NEFALate, and P4Late levels were significantly lower in cows that were given feed supplements compared to the control group (*p* < 0.01). Conversely, GluLate levels were notably higher in the treated cows (Figure 2A; *p* < 0.01). The correlations between different blood metabolite variables are illustrated in Figure 2B. The data indicates that GluLate has a positive relationship with BUNMid and TPLate, but demonstrates weak to negative associations with other metabolites. The correlation coefficient for TGLate and NEFALate is +1, meaning they show identical correlation values with other traits. Both these variables also exhibit strong correlations with BUNLate, P4Mid, and P4Late concentrations. Additionally, P4Late shares high correlations with P4Mid, NEFALate, ChoMid, and ChoLate. Correlations between other pairs of pregnant cow blood metabolites are displayed in Figure 2B.

### 3.2. Effects of mid-to-late pregnancy supplemental nutrition in Hanwoo cows on different parameters in slaughtered neonatal calves

#### 3.2.1. Anatomical metrics of gastrointestinal tissues.

**Figure 3:**
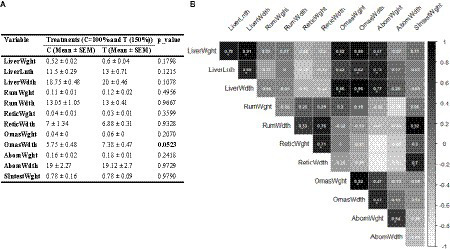
**(A)** Impact of mid-to-late pregnancy nutritional supplementation on neonatal calf gastrointestinal tissue metrics; **(B)** Correlations between these metrics. Correlation coefficient values higher than 0.5 are marked with an “*” sign.

The t-test analysis indicated that the OmasWdth values in the T group were marginally higher than those in the control group (*p* = 0.053). However, other measurements did not show significant differences between the two groups (Figure 3A). Additionally, the correlation analysis indicated a strong association between OmasWdth and both liver measurements and OmasWght (Figure 3B). Correlation values for other variable pairs can also be found in Figure 3B.

Table 2 illustrates the effects of nutritional supplementation during mid-to-late pregnancy on the muscle fiber measurements and the metrics of their gastrointestinal papillae and villi of neonatal calves. The results indicated that the count and diameter of fibers in the loin and round muscles did not exhibit any statistically significant differences between the two groups of slaughtered calves (*p* > 0.05). In contrast, the lengths of papillae and villus in the rumen (RumPapilLnth; *p* < 0.05) and small intestine (SIntestiVillnth; *p* < 0.01) were significantly higher in the treated group than in the control group. However, there were no significant differences observed in the count of papillae (SIntestiVillnth) in the rumen, nor in the lengths and counts of papillae in the reticulum (RetPapilLnth and RetNmbrPapil; *p* > 0.05) and omasum (OmasPapilLnth and OmasNmbrPapil; p > 0.05).

**Table 2:** Impact of mid-to-late pregnancy nutritional supplementation on newborn calf muscle and gastrointestinal papillae and villi metrics.

Variable	Treatments (C = 100% and T (150%))		*p*-value
	C (Mean ± SEM)	T (Mean ± SEM)	
loinFbrDmtr	25.81 ± 1.54	27.54 ± 1.63	0.471
loinFbrNmbr	25.06 ± 5.34	18.17 ± 4.66	0.369
RoundFbrDmtr	26.21 ± 0.97	28.51 ± 1.79	0.314
RoundFbrNmbr	26.21 ± 4.45	17.41 ± 4.71	0.224
AbomMucThick	482.68 ± 48.4	516.76 ± 32.09	0.582
RumPapilLnth	848.54 ± 65.56	1147.1 ± 77.15	**0.026**
RumPapilNmbr	42.73 ± 3.19	42.41 ± 1.42	0.931
RetPapilLnth	2687.96 ± 115.64	2950.32 ± 453.97	0.610
RetNmbrPapil	17.42 ± 1.77	32.4 ± 10.47	0.248
OmasPapilLnth	10611.7 ± 1981.95	7197.66 ± 751.2	0.185
OmasPapilNmbr	8.37 ± 0.98	9.35 ± 0.83	0.475
SIntestiVillnth	738.89 ± 84.23	1260.33 ± 80.99	**0.004**

#### 3.2.2. Morphometric analysis of calf external body parameters

**Figure 4:**
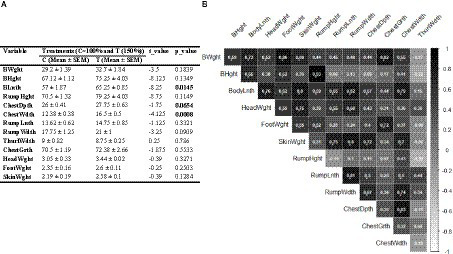
**(A)** Effect of nutritional supplementation during mid-to-late pregnancy on calf external body parameters. **(B)** Correlation between different pairs of these metabolic parameters. Correlation coefficient values higher than 0.5 are marked with an “*” sign.

Figure 4 illustrates the impact of nutritional supplementation during mid-to-late pregnancy on morphometric measurements of body and various tissues in neonatal calves, along with their correlation coefficient values. There were no significant differences in body weight (BWght) and height (BHght) between the treated and control groups (*p* > 0.05). However, calves in the treated group exhibited a notably longer body length (Blnth) than those in the control group (*p* < 0.05). Additionally, the treated group had a significantly greater ChestWdth value compared to the control group (*p* < 0.01). While ChestDpth (*p* = 0.065) and RumpWdth (*p* = 0.09) measurements were marginally higher in the treated group, there was no statistically significant difference. No significant differences were noted for the other parameters between the two groups (*p* > 0.05; Figure 4A). Correlation analysis revealed that Blnth and ChestWdth had strong positive correlations with all other parameters. Notably, among all parameters, only ThurlWdth displayed low or negative correlations with the others, as depicted in Figure 4B.

#### 3.2.3. Carcass characteristics of neonatal calves

Table 3 displays the influence of nutritional supplementation during mid-to-late pregnancy on neonatal calves’ carcass characteristics. No significant differences in the carcass parameters were observed between the two groups of calves (*p* > 0.05). While most parameters in the treated group were slightly higher, this was not the case for MeatPerc, BonPerc, PlatePerc, and BotRoundPerc compared to the control group.

**Table 3:** Effect of mid-to-late pregnancy nutritional supplementation on carcass characteristics of newborn calves.

Variable	Treatments (C = 100% and T (150%))		*p*-value
	C (Mean ± SEM)	T (Mean ± SEM)	
CarcWght	14.93 ± 0.74	17.25 ± 1.14	0.1485
MeatPrec	60.14 ± 1.08	60 ± 0.21	0.9045
FatPerc	4.91 ± 1.18	5.9 ± 0.7	0.5029
BonPerc	34.95 ± 0.74	34.1 ± 0.53	0.3911
TendloinPerc	2.45 ± 0.26	2.71 ± 0.1	0.4082
SirloinPerc	6.74 ± 0.52	7.28 ± 0.78	0.5894
StriploinPerc	1.76 ± 0.06	1.94 ± 0.09	0.1606
ChuckPerc	4.6 ± 0.57	5.14 ± 0.15	0.4102
PlatePerc	8.73 ± 0.44	8.67 ± 0.57	0.9290
RoundPerc	7.2 ± 0.32	7.38 ± 0.53	0.7758
BotRoundPerc	11.32 ± 0.42	11.04 ± 0.99	0.8107
ShankPerc	9.6 ± 0.58	9.77 ± 0.74	0.8581
BrisketPerc	10.2 ± 0.82	10.89 ± 0.59	0.5211
RibPerc	4.53 ± 0.17	5.31 ± 0.26	0.0523

#### 3.2.4. Blood metabolites of neonatal calves

Table 4 presents the impact of nutritional supplementation during mid-to-late pregnancy on select blood metabolites in slaughtered neonatal calves. The data indicated that GPT levels were significantly lower (*p* < 0.05) in the treated calves compared to the control group, while the decrease in GOT levels was not statistically significant. Furthermore, the concentrations of glucose (Glu), triglycerides (TG), total protein (TP), and phosphorous (IP) were higher in the treated group, but these differences were not statistically significant. Conversely, the levels of albumin (Alb), cholesterol (Cho), BUN, and NEFA were lower in the treated group compared to the controls, but again, these differences did not reach statistical significance.

**Table 4:** Effect of mid-to-late pregnancy nutritional supplementation on some blood metabolites of slaughtered newborn calves.

Variable	Treatments (C = 100% and T (150%))		*p*-value
	C (Mean ± SEM)	T (Mean ± SEM)	
Alb	2.1 ± 0.15	2 ± 0.3	0.8016
Glu	56.67 ± 34.37	99 ± 4	0.3430
Cho	25.67 ± 4.98	24.5 ± 10.5	0.9320
TG	11.67 ± 9.17	21 ± 20	0.7264
BUN	14.03 ± 1.66	13.3 ± 4.7	0.9031
TP	5.13 ± 0.68	5.25 ± 0.85	0.9236
GOT	72.33 ± 10.53	55.5 ± 12.5	0.3977
GPT	11 ± 0.58	7.5 ± 0.5	**0.0213**
IP	5.93 ± 0.37	8.75 ± 0.95	0.1706
NEFA	0.49 ± 0.18	0.42 ± 0.08	0.7292

## 4. Discussion

Nutritional management of pregnant cows during the mid-to-late pregnancy period exerts a substantial influence on both the health and productivity of the cow, as well as the physiological outcomes of the calf. This interplay between maternal nutrition and fetal outcomes has been a center of attention in livestock development, influencing herd health, beef quality, and yield (3). Historically, maternal dietary regimes during pregnancy have been understood to shape the physiological framework and performance metrics of the progeny (1, 2). In this study, we delved into the effects of feed supplementation mid-to-late pregnancy period on performance metrics and metabolic parameters in Hanwoo cows and on some gastrointestinal metrics, the body measurements, the carcass characteristics and blood metabolite profiles of their neonatal calves.

In terms of performance, significant improvements observed in PregP and BCS in cows receiving supplemental feeding emphasize the benefits of nutrition during the pregnancy period under study. These findings align with the observations that BW changes during late pregnancy reflect the balance between nutrient intake and nutrient requirements (25). However, the absence of statistically significant differences in BW and FCR suggests that while supplemental feeding may improve the overall physiological health of cows, as reflected by BCS, it does not markedly influence their overall BW or FCR. This observation corroborates the findings by Mulliniks et al. (25), which stated that cows maintaining or losing BW during later pregnancy were initially heavier. The inverse relationship between BCS and FCR could be indicative of energy being utilized more for maintenance and pregnancy than weight gain, especially in cows with better BCSs (25).

Our data further showed a rise in GluLate levels and a significant decrease in TGLate, NEFALate, and P4Late in cows given supplemental feed, emphasizing the significance of Glu as a predominant energy substrate for pregnant animals to maintain tissue and organ function and to foster fetal development (26). The increase in Glu levels can be attributed to research indicating that when energy intake surpasses prepartum requirements, it results in elevated prepartum blood Glu levels (27). Elevated Glu levels can be attributed to the high energy diet’s starch-rich components that promote Glu production (22). Furthermore, decreased NEFA levels suggest the animals were not in a catabolic state, tapping into fat reserves, a response observed in previous studies under nutrient-restricted conditions (26). P4, vital for initiating and sustaining pregnancy (24), showed decreased levels in our treated cows. Previous studies indicated that P4 levels were contingent upon corpus luteum health and can be influenced by diet (24). Our study’s reduced P4Late levels might signify the cows’ dietary influences and potential effects on conceptus growth and maintenance.

Several literature reports have emphasized the significance of maternal nutrition during late pregnancy on progeny performance. Underwood et al. (29) reported marbling scores in the offspring remained consistent irrespective of the quality of maternal pastures during late pregnancy. The pivotal influence of carbohydrate sources on adipose tissue accumulation has been emphasized by studies suggesting Glu contributes majorly to intramuscular fat, whereas acetate mainly serves subcutaneous fat (11). However, some research contradicts our findings. For instance, while we noticed significant alterations in BCS and BW with supplemental feeding, some studies found no discernible influence of varied fatty acid profiles or feed supplementation type/quantity on BW and BCS during late pregnancy (30, 31). Additionally, Shao et al. (31) found that BCS remained consistent or even increased with feed supplementation strategies, supporting our results. The consistency of BCS over time suggests its accuracy as a representation of prolonged nutritional status, contrasting with BW fluctuations (32).

Intriguingly, our findings exhibited strong correlations between specific blood metabolites. This is consistent with previous literature. For example, elevated NEFA levels in the blood typically imply a negative energy balance and have been inversely correlated with ADG and nutrient consumption (33). NEFA and Glu dynamics play a vital role in determining an animal’s energy balance and could have profound implications for the offspring’s metabolic health and overall performance.

The marginal increase in OmasWdth in the treatment group, as indicated by the t-test (*p* = 0.053), suggests a potential influence of maternal nutrition on the gastrointestinal development of the offspring. This is particularly notable as the omasum plays an essential role in the absorption of nutrients in ruminants (2). This trend aligns with the strong association observed between OmasWdth and both liver measurements and OmasWght. Liver functionality is paramount for metabolite processing and overall health in cattle (1), suggesting that a slight variation in this dimension might impact the metabolic efficiency of the calves.

The lengths of papillae and villi in the rumen and small intestine displayed notable disparities, echoing the findings of Chen et al. (21) that emphasize the role of maternal-origin metabolic byproducts in fetal tissue development. While our observations corroborate the broader concept of fetal programming, which underscores the integral relationship between maternal nutrition and neonate physiology, our data presents a contrasting view to Mulliniks et al. (30).

The evident variation in OmasWdth among offspring from the supplemented cows reinforces the significant impact of maternal nutrition on gastrointestinal development in the offspring (2). These nutritional adaptations, potentially impact the calf’s postnatal feed efficiency, nutrient uptake, and overall growth trajectory (3). Further, the increased lengths of papillae and villi in the rumen and small intestine of the treated group underscore the potential benefits of supplemental nutrition in enhancing gut surface area, a factor that could significantly improve nutrient absorption during postnatal life (5).

Morphometric analysis in our study provides an understanding of the influence of maternal nutrition on neonatal calf morphometric. The experimental group exhibited an increased Blnth and enhanced ChestWdth, indicating that supplementation during the mid-to-late gestational period might influence these parameters. These observations partly align with research from Chen et al. (21), which found calves from the high energy diet group displayed notable improvements in birth weight, body height, and thoracic girth. Similarly, findings from literature underscore the pivotal role maternal nutrition plays in determining calf performance and carcass characteristics (1, 11, 34, 35). Despite these morphometric variations, it’s worth noting that birth weight remained unaffected, consistent with the findings of Funston et al. (1). This suggests that while nutritional interventions during pregnancy might not significantly alter overall neonatal growth parameters, they have the potential to influence specific growth indices.

Our investigation into carcass characteristics revealed that, despite observed morphometric variations, these parameters remained largely unaffected by maternal nutritional supplementation. Specifically, factors like MeatPerc, BonPerc, PlatePerc, and BotRoundPerc in the treated group were slightly elevated but showed no significant differences between groups. These observations align with previous findings which indicated that offspring of mothers with different nutritional profiles during pregnancy did not show notable differences in metrics such as hot carcass weight, intramuscular fat percentage, or marbling score (11, 35). Our data seems to agree with Du et al. (4), suggesting that *in utero* nutritional interventions might redirect nutrients toward specific tissue types or growth processes, particularly regarding muscle-to-fat proportion during mid-pregnancy. Although our findings and those on muscle fiber metrics suggest that *in utero* nutritional strategies, especially during mid-to-late pregnancy, may not be the primary determinant for neonatal calf carcass quality, the metabolic imprinting hypothesis proposed by LeMaster et al. (22) underscores the possibility of long-lasting effects on metabolism, physiology, and productivity. As such, the long-term ramifications of these nutritional interventions merit deeper exploration.

The blood metabolite data emphasize the complex consequence of maternal nutrition on calf health and metabolism. A marked decline in GPT levels in calves from the treated group has been observed, suggesting potential implications on liver function or health. Given that GPT is frequently regarded as an indicator of liver stress or injury (22), the observed decrease may suggest an enhanced functionality or reduced stress of liver, suggesting the pivotal importance of liver in the overall metabolic health, growth, and productivity of livestock. While other blood metabolites like Glu, TG, Alb, Cho, and NEFA showed no significant statistical differences between the groups, it’s important to consider their potential influence on energy metabolism and growth in calves’ postnatal life (23). According to the findings of Konigsson et al. (33), elevated NEFA levels, which are often indicative of a negative energy balance, can be inversely associated with daily gains and nutrient intake. The present observations also resonate with (21)'s emphasis on the crucial role of maternal energy density during the late period of pregnancy on the antioxidative capacity of neonates. Nevertheless, the non-significant variations in several metabolites in this work suggest the necessity for more extensive studies to validate these initial findings.

## 5. Conclusion

Supplemental feeding during mid-to-late pregnancy in Hanwoo cows provides significant benefits, improving cow BCS, influencing blood metabolites, and supporting neonatal calf gastrointestinal development. However, certain calf muscle and morphometric traits such as loinFbrDmtr, RoundFbrNmbr, ChestGrth, HeadWght and, etc. remain unchanged. This research emphasizes the importance of maternal nutrition on livestock offspring’s future performance. Notably, the full extent and implications of these findings warrant further in-depth analysis. Despite the limited sample size, the data underscores the importance of nutritional strategies during pregnancy for the beef industry, especially in the case of breeds like Hanwoo. Further extensive research is essential to gain a deeper understanding of maternal nutrition’s impact on calf health and productivity, which is crucial for the refinement of management protocols. Future studies focusing on molecular and genetic markers could deepen insights into fetal development impacts at molecular and cellular levels.

## Data availability statement

The original contributions presented in the study are included in the article/Supplementary material, further inquiries can be directed to the corresponding authors.

## Ethics statement

The animal study was approved by Institutional Animal Care and Use Committee of the National Institute of Animal Science (NIAS), under reference number NIAS 2020-095. The study was conducted in accordance with the local legislation and institutional requirements.

## Author contributions

MP: Conceptualization, Data curation, Formal analysis, Investigation, Methodology, Project administration, Writing – review & editing. BS: Formal analysis, Methodology, Software, Validation, Visualization, Writing – original draft, Writing – review & editing. UK: Data curation, Investigation, Methodology, Writing – review & editing. JW: Data curation, Investigation, Methodology, Writing – review & editing. S-HC: Investigation, Methodology, Writing – review & editing. SJi: Investigation, Methodology, Writing – review & editing. SKa: Investigation, Methodology, Writing – review & editing. SM: Investigation, Writing – review & editing. K-HU: Investigation, Writing – review & editing. KJ: Investigation, Writing – review & editing. HK: Investigation, Writing – review & editing. NK: Investigation, Methodology, Writing – review & editing. SKi: Investigation, Writing – review & editing. SJa: Funding acquisition, Project administration, Writing – review & editing. H-JL: Conceptualization, Data curation, Funding acquisition, Resources, Supervision, Validation, Visualization, Writing – review & editing.
